# Variation in the Protein Composition of Human Milk during Extended Lactation: A Narrative Review

**DOI:** 10.3390/nu10081124

**Published:** 2018-08-20

**Authors:** Sergio Verd, Gemma Ginovart, Javier Calvo, Jaume Ponce-Taylor, Antoni Gaya

**Affiliations:** 1 Pediatric Division, Department of Primary Care, La Vileta Surgery, 07013 Palma de Mallorca, Spain; jaume.ponce@gmail.com; 2 Neonatal Unit, Department of Pediatrics, Hospital de la Santa Creu i Sant Pau, 89 Sant Quinti St, 08041 Barcelona, Spain; gginovart@santpau.cat; 3 Blood and Tissue Bank of the Balearic Islands (Milk Bank), 07004 Palma de Mallorca, Spain; jcalvo@fbstib.org (J.C.); agaya@fbstib.org (A.G.); 4 Cell Therapy and Tissue Engineering Group (TERCIT), Balearic Institute of Medical Research (IdISBa), Valldemossa Rd., 07120 Palma de Mallorca, Spain

**Keywords:** milk bank, premature infant, breastfeeding, human milk physiology, lactation chemistry, nutritional requirements

## Abstract

The aim of this review is to evaluate changes in protein parameters in the second year postpartum. There is considerable agreement among authors about the declining trend of human milk protein concentrations, but most research on protein content in breast milk focuses on the first year of life and comes from developed countries. Whereas this is the case for exclusive breastfeeding or for breastfeeding into the first year of life, the opposite applies to weaning or extended breastfeeding. This review is predominantly based on observational epidemiological evidence and on comparative research linking breast milk composition with cutting down on breastfeeding. Studies dating back several decades have shown an increase in the proportion of immunoglobulins, lactoferrin, and serum albumin during weaning. According to the limited data available, it seems likely that the regulation of milk protein composition during involution can be ascribed to alterations in tight junctions. In studies on humans and other mammalian species, offspring suckle more from mothers that produce more dilute milk and the increase in milk protein concentration is positively correlated to a decrease in suckling frequency during weaning. High milk protein contents were first reported in nonindustrial communities where breastfeeding is sustained the longest, but recent papers from urbanized communities have taken credit for rediscovering the increase in protein content of human milk that becomes evident with prolonged breastfeeding. This review presents an overview of the changes in breast milk protein parameters in the second year postpartum to enable milk banks’ practitioners to make informed nutritional decisions on preterm infants.

## 1. Introduction

The nutrient composition of human milk can be strongly influenced by the stage of lactation. These changes are consistent with consuming decreasing quantities of milk and ingesting other foods throughout the course of lactation as the infant matures [1]. Studies dating back 40 years have shown that milk composition during weaning from extended nursing is different from that of exclusive breastfeeding [2]. Whereas the concentration of lactose decreases fivefold during mammary involution, the concentration of proteins increases sixfold during this period [3]. It is worth highlighting the high content of antibacterial proteins during mammary involution, which provides the breast with protection against infection at a time when milk stasis favors bacterial growth, and would also favor those ill toddlers who return to the breast during weaning [4]. More importantly, the increased protein concentrations observed in prolonged breastfeeding suggest that donor milk from the weaning period may be more appropriate than early donor milk to meet the nutrient requirements calculated to achieve adequate growth rates among preterm infants when a mother’s own milk must be augmented with donor milk.

Coincident with the lack of public acceptance of extended breastfeeding, these findings have been ignored for decades. Numerous studies have evaluated protein and amino acid levels in human milk. There is now considerable agreement among researchers that the protein concentration in human milk undergoes a sustained decline over the first year of lactation. Three recent studies that evaluate the change in protein content of breast milk during infancy concluded that the reduction in protein over the first year of lactation exhibits a linear pattern [5,6,7]. Conversely, since 2016 two groups have studied longitudinal changes in human milk composition beyond one year of age and describe the total protein concentration of human milk increasing steadily in the second year postpartum [4,8]. It should be noted that these surveys were undertaken when research has found a continued increase in the percentage of beyond-20-month-old children still breastfeeding [9]. Western nations appear to have been outliers, whereas this is an ordinary weaning age in traditional societies [10].

The variation of protein content in breast milk between birth and the first year postpartum has been evaluated in a variety of studies, with results that are in close agreement. However, when it comes to milk composition during the later stages of lactation, there is much less information available. Most studies on milk composition beyond month six are set in developing countries, where extended breastfeeding has been the rule [11]. Based on the aforementioned issues, opening up a dialogue on the true longitudinal evolution of breast milk protein content seems timely. Thus, we conducted this review to evaluate changes in protein parameters beyond the first year of lactation. The aim is to revisit the forgotten topic of protein content in human milk in the second year postpartum to enable milk banks to make informed nutritional decisions for preterm infants. We propose a more comprehensive description of milk protein content that captures a diversity of results from major fields of research.

We begin by discussing the regulation of mammary tight junctions. Both local factors, such as TGF-beta, and systemic factors, such as prolactin and cortisol, regulate the movement of material through the paracellular pathway [12,13,14]. Numerous biomarkers affect the blood–milk barrier. However, it remains unclear what induces less permeable tight junctions and no biomarkers are used as a diagnostic tool in clinical settings.

## 2. Methods

Only published studies with well-described methodology and widely accepted data collection methods were reviewed. Commentaries and protocols were not included in the analysis.

The following electronic databases were considered to be the most relevant for the topic and were searched: Cochrane Library, MEDLINE (PubMed), Embase and CINAHL (EBSCOhost). A health sciences librarian was consulted for the database search strategy. The databases were searched until the week starting 25 March 2018. Table 1 outlines the search strategies and key terms used. Searches were limited to mammal species and results were restricted to English-, French-, and Spanish-language publications. The initial search strategy was developed for MEDLINE and then adapted to the other databases. Further studies were found using the reference lists of the studies initially identified.

## 3. Pathophysiology

### 3.1. Tight Junction Regulation

Neville [3] reminds us that, in 1935, Widdows et al. [15] noticed that “A comparison of the relative proportions of the main ingredients of the fluid secreted by the mammary gland before and after parturition shows that a true difference in secretory activity is involved and not merely a difference in concentration.” Subsequent studies have shown a fascinating transition from a highly permeable epithelium in pregnancy to the tight epithelium of lactogenesis [16] and, finally, to junctional complexes that open again during weaning. This occurs when milk volumes fall below 66% of baseline volumes, when experimental subjects wean their infants gradually by reducing the frequency or the duration of breastfeeding by approximately one-third each month [17]. Tight junctions are important in preserving the three-dimensional structure of epithelia. In the mammary gland junctions between adjacent epithelial cells are formed during lactogenesis and are key to maintaining milk secretion. They prevent the paracellular transport of ions and small molecules between the blood and milk compartments. It seems likely that the delay between the closure of the tight junctions and the onset of copious milk secretion prevents interstitial edema. If large amounts of lactose and proteins were to appear in the mammary lumina while there was still continuity between the milk and the extracellular compartments, sufficient milk components might enter the interstitium to produce substantial breast engorgement. Intact tight junctions are important at the start of lactation, whereas tight junction integrity is compromised at preterm birth, during mammary involution, and during periods of mammary inflammation [18]. It has been hypothesized that the increased proteolytic activity in mastitis and in the immature mammary gland of preterm mothers results from increased barrier permeability. It has also been hypothesized that incomplete closure of tight junctions in these cases underlies an increased leakage of proteases into the milk. Compared with the full-term infant, the preterm infant is poorly equipped for protein digestion and the increased proteolytic activity in the preterm infant may compensate for their lower protein digestion within the small intestine [19].

Current knowledge on leaky tight junctions supports the idea that the changes in the composition of the milk of lactating women are strongly influenced by the weaning process, the dietary transition from exclusive breastfeeding to non-milk food. In weaning subjects the overall concentration of protein increases by 1.6-fold compared to the non-weaning subject, but the protein concentration in the milk from the unsuckled breast is 2.8-fold higher than in the milk from the normally suckled breast of the same woman [20]. These results may indicate a progressive loss of secretory activity by the mammary gland during weaning, but researchers recorded daily changes in the composition of the milk of only four lactating women. During rapid weaning, when milk volume falls below 300 mL/day, the protein content increases to 20% [21]. Concerning gradual weaning, protein concentrations have been measured over a three-month weaning period. The final concentration of protein was 142% of the baseline value. This increase in the concentration of protein over time was statistically significant and can be described linearly. However, there is also a significant quadratic trend with the rate of change increasing in the latter phase of weaning. The accelerated changes in protein concentrations observed from week 8 of weaning may reflect an increase in glandular involution [18]. The major observations from the detailed analysis of milk composition during weaning are significant increases in electrolytes and protein, and a decrease in the lactose concentration in milk with a progressive decline in milk volume. According to the reviewers of this topic, these observations support that a reduction in the mammary gland’s synthetic activity, an increase in paracellular transport processes over the previously predominant transcellular mechanisms, and changes in water transport appear to govern milk composition changes during mammary involution [22].

### 3.2. Milk Protein Composition

Milk contains hundreds of intact proteins. It is useful to consider some of the milk protein components separately. There are differences in the rate at which the concentration of mammary proteins increase during involution. The proteins synthesized by the alveolar cells (*α*-lactalbumin and caseins) increase proportionally less than lactoferrin, serum albumin and the immunoglobulins. Hartmann has determined the mean changes in the concentration of most protein components during involution after 11 months of lactation [2]. Protein values were obtained 7 days before and 42 days after the termination of breastfeeding. The concentration of *α*-lactalbumin increased 2-fold over the first 21 days and then decreased slightly over the next 21 days, whereas over the 42 days after termination of breastfeeding, the concentration of IgG, IgM and IgA increased 8-fold, 20-fold and 5-fold, respectively. The increased content of lactoferrin and the immunoglobulins during involution provides protection at a time when breast engorgement favors bacterial growth. The high content of antibacterial immunoglobulins would also favor ill infants who return to the breast during weaning. Other authors have also observed increases in antimicrobial proteins of human milk (lysozyme, lactoferrin and IgA) in the second year of lactation [4].

To summarize, an increase in the proportion of immunoglobulins, lactoferrin and serum albumin, and a decrease in the proportion of caseins and *α*-lactalbumin was observed during weaning. Conversely, changes in protein concentration and composition in the 96 h postpartum reflect a dramatic decrease in the rate of secretion of immunoglobulins, which overlaps with an increase in the rate of secretion of caseins and *α*-lactalbumin. According to the limited data available, it seems unlikely that the regulation of milk protein composition during lactogenesis can be ascribed to alterations in tight junctions and therefore it is not similar to cellular mechanisms operating during involution.

### 3.3. Suckling Patterns

In horses, offspring suckle more from mares that produce more dilute milk [23]. Similarly, the increase in milk protein concentration during weaning is positively correlated to the decrease in the suckling frequency of the infant [20].

Both the duration of lactation and the degree of suckling appear to govern the progression of mammary gland involution [22]. Due to the relatively low protein characteristics of human breast milk, newborn babies are considered as a continuous contact, frequent suckling species. Alternatively, other mammal species that produce highly-concentrated milk are called spaced feeders because they leave their young in nests during long separations; these babies avoid starvation if they suckle fast and efficiently very rich milk; a good example is the rabbit, which produces milk that is 18.3% fat and 13.9% protein [24]. Cross-cultural surveys of infant feeding show that, within many nonindustrial populations, neonates are with their mother almost constantly throughout the day; in fact they are held by the mother usually for more than 50% of the time. Young infants are allowed to nurse whenever they want and for as long as they want; it is considered imperative that a woman feeds her baby every time she cries [10]. In hunter–gatherer societies infants are nursed every hour [25]. In other traditional societies parents do not match this extreme pace [26], but in a survey that included nomadic and settled agricultural peoples anthropologists found that on demand feeding was the rule (25 out of 25 groups) [27]. Westerners tend to space out the feedings such that babies get fed after intervals of 3–4 h, but this schedule is not consistent with the basic physiology of human breastfeeding nor with the behavior of other members of our family tree. Continual feeding is the strategy of choice among bonobos, chimpanzees, and gorillas; human milk is relatively low in protein (1%) and human infants suckle at a slow pace that is typical of continual feeders. Frequent suckling reduces human milk protein content as well as female fecundity, and hence longer periods of exclusive breastfeeding can lead to fewer children born per woman. When non-breastmilk foods are first introduced, frequent suckling and consumption of breastmilk decrease. During weaning this loss appears to be directly related to the compositional changes observed in human milk, i.e., higher protein concentration.

The subject of parallels between ontogeny and phylogeny is still one of the great themes of evolutionary biology. It applies to this topic, yet infrequent suckling has been shown to be associated with higher milk protein content among spaced feeder mammals and also when continual feeder mammals nurse less frequently their babies, i.e., during weaning.

## 4. Variation in the Protein Content of Milk: A Comparative Perspective

The potential gain in knowledge from comparative studies is great. Studies on different mammalian species can provide additional insight into the regulation of milk composition. Lactation has evolved to meet the demands of different species. Breastmilk provides offspring with a safe source of nutrients together with immunological protection, but weaning is necessary to meet the growing requirements of offspring and constitutes a major determinant in the survival of mammals [28]. The nutrient composition of milk not only varies dramatically across species, but can also be strongly influenced by the length of lactation. The literature in this field shows which factors contribute to between- and within-species variations in milk constituents and provides an evolutionary perspective on the maternal investment in lactation. Upon examination of the milks of hundreds of species of mammals, previous research has shown considerable variation in their overall composition. For example, primate milk, including humans, is relatively devoid of casein (0.1%), whereas pig milk contains 4% casein [29]. Additionally, there are fewer differences in the composition of colostrum than in the contents of milk by the end of lactation. In particular, early lactation stages deliver ample calcium and phosphate due to the extensive bone and tooth development that occurs before the transition to independent feeding. The fat content of milk may be negligible during early lactation but may be as high as 60% at late lactation [30].

Milk bears substantial evolutionary pressures: it costs energy to the mother and, therefore, must have positive effects on the neonate. Lactation represents the greatest postnatal energetic expenditure for mammal females [31], and is directly influenced by the mother’s physical condition. The relationship of body mass to milk macronutrient composition has been extensively considered [32,33]. Especially, very young mothers have fewer bodily reserves available to face simultaneously the costs of lactation and of their own growth [34]. Other factors contributing to variability in milk composition include parity, diet, offspring sex, and maternal health status. In case of significant sexual dimorphism in infant mass, mothers of sons produce milk of higher energy density, with a lower milk yield, when compared to mothers of daughters [35]. There are optimal strategies for food supplementation during lactation for many mammals. Most large mammals lactate for a long period of months or years, produce milk with relatively low energy content and wean their pups gradually. In contrast, other mammal species lactate for brief periods of one month or less, produce energy-rich milk and wean their pups abruptly. The mother tries to reduce her energy expenditure as much as possible, while the offspring tends to obtain as much building material as possible [36].

In spite of less time spent nursing, increasing protein concentrations are present in the milk of nonhuman primates, starting around the age at which weaning begins for these species. This is a period of developing independence from the mother, which includes solid food feeding and rapid growth [37]. The same applies to marsupials. The postpartum lactation cycle of marsupials is divided into three phases. At birth, the antiracial young climbs into the pouch and attaches to the teat of one mammary gland. During phase 2 of lactation the young remains in the pouch and shifts from permanent attachment to the teat to intermittent, but frequent sucking. Phase 3 of lactation is characterized by the young beginning to exit the pouch, to eat grass, and to suck at less frequent intervals [38]. A study performed with marsupials (*n* = 35) shows that on transition from lactation phases 1 or 2 to phase 3, the gross milk composition changes markedly, and the protein content of milk significantly increases.

Milk also enhances the survival of offspring by promoting immunological competence. In this regard, the rising concentrations of bioactive proteins, i.e., less digestible and of less nutritional value [39], that occur as lactation progresses are likely a physiological response to the diverse environmental demands of the hatchlings as they progressively acquire independence. Developmental changes in composition of milk intake have been followed for few nonhuman species [40,41], but similar relations of increased macronutrient concentration during declining lactation or mammary involution have been observed across species [2]. It applies, among others, to rats and cows [42,43]. Human milk follows similar trends for protein concentrations associated to gradual or abrupt weaning. Comparative research has also evaluated the variability of milk composition for animals with different kid suckling periods. The highest milk protein content is obtained from goats when kids are weaned on the first day of life [44].

Transition to nonmilk food, which supplements the mother’s milk in the course of weaning, exposes nursing pups to exogenous pathogens and energy shortfalls. The inverse relationship between the fall in mammary secretion and the increase in milk protein components suggests the existence of similar mechanisms, in humans and other species, that allow the passage of proteins through the blood–milk barrier in significant amounts [17] at the age when weaning begins. It may be that breastfeeding protein requirements are at their highest during this time period to support both the immune system function and the metabolic demands of independent ambulation.

## 5. Epidemiological Evidence

For more than 40 years the prevailing viewpoint has been that concentrations of proteins in human milk are highest in the first weeks of lactation and then continuously decline. The extent to which protein content in breast milk changes over time has been evaluated in a variety of observational studies [45,46]. According to Lonnerdal et al. [5] the protein decline in infancy follows a logarithmic model and according to Kreissl et al. [7] the protein content from mothers of young infants shows a negative correlation (*r* = −0.42) with the day of lactation. Diminishing rates of physical growth during infancy are a possible explanation for decreasing protein requirements during the later stages of lactation [30,47]. Aforementioned citations have overlooked other components of scientific contribution that emphasize the increase of human milk protein concentration in the second year postpartum. The pioneer studies in this area reported that when weaning, there is a huge increase of antibacterial proteins in human milk [2]. Two recent papers have taken credit for rediscovering the increase in protein content of human milk with prolonged breastfeeding [4,8]. This research was made possible because lactating women were recruited using local parenting groups of extended breastfeeding that communicated via social networks.

As expected, the first surprising findings on high milk protein contents come from poor communities where breastfeeding is most common and is sustained the longest [48]. In 1952, Jelliffe reported high levels of protein content (1.79 g/dL) among Nigerian children (*n* = 88), and in 1958 Gopalan noticed in India that a fall in protein concentration has a correlation to an increased output of milk [11]. Thereafter, similar data have been collected from affluent urbanized communities. Michaelsen et al. [49] described the variation in protein concentration of human milk samples from the Copenhagen Milk Bank. Between June 1984 and December 1986, 2554 samples from 244 donor mothers were analyzed. Donors were supposed to breastfeed their child for at least 4–6 months. No drip milk was collected. There were no limitations to the length of time during which the mother could continue to donate milk. The protein content decreased during the first eight months, more importantly it decreased with increasing amounts of milk donated to the milk bank. It was followed by an increase of protein content thereafter. The authors describe the negative correlation between protein content and milk volumes, a 10-L decrease in the monthly volume predicted an increase in protein content of about 10%. They conclude that by continuous monitoring of macronutrient concentration of milk samples it is possible to collect milk with a high protein content, especially suitable for preterm infants. In 1993, the DARLING Study was able to document total nutrient intakes and growth patterns of breast and formula-fed infants during the first 18 months of life. Ninety-two mothers who planned to continue breastfeeding for a minimum of 12 months were initially recruited [21]. Similarly to Michaelsen et al. [49], they found that a decrease in the milk volume predicted an increase in protein content. The volume of breast milk produced at six months and at nine months was negatively associated to human milk protein concentration. No other selected variable contributed significantly to the variability in protein concentration. The authors had to acknowledge that negative associations between milk volume and protein concentrations were not easily explicable. Conversely, all reviews on longitudinal evolution of macronutrients in breast milk have included studies reporting outcomes mainly from exclusively breast-fed infants and have excluded reports on weaning infants that show how human milk protein concentration is negatively related to milk volume and positively related to nursing frequency. In their evaluation of human milk protein content, Lonnerdal et al. [5] found a total of 43 original articles published between 1953 and 2011, and included 34 of them in their analysis. Their dataset demonstrates that the dynamic evolution of human milk contents exhibits a logarithmic decay from birth to year one. On the other hand, a recent review of 16 reports on protein and amino-acid composition of human milk found a range of 8.5–11.9 g protein/dL milk when the authors included samples from lactation days between 1 and 188, whereas the mean protein level for samples from lactation days between 46 and 297 rose to 12.6 g/dL [50].

These reviews do not overshadow recent reports on continued increase of human milk protein concentration throughout the second and third years of life. In 2017, Czosnykowska-Lukacka et al. [8] compared the macronutrient composition of 41 samples of ordinary mother’s own milk with 38 samples from standard donors, and with 15 samples from mother breastfeeding for two years. Mother’s milk after two years of lactation contained a macronutrient concentration of proteins that was, on average, two and a half times higher than an ordinary mother’s own milk and standard donor milk. The authors conclude that long term breastfeeding mother’s milk may provide the best donor milk for preterm infants. Also in 2017, Perrin et al. [4] described longitudinal changes in human milk composition in the second year postpartum. The purpose of their study was to support the development of evidence based guidelines regarding how long lactating women can donate milk to a milk bank. They recruited 33 women that had given birth to a full-term infant who was 9–11 months old at the time of enrollment, and that intended to breastfeed until their child was at least 18 months. The total protein concentration as well as lysozyme, lactoferrin, and IgA contents increased longitudinally in the second year postpartum. The authors consider these changes throughout the course of lactation to have implications for the implementation of more effective preterm infant feeding policies.

## 6. Conclusions

This review has provided an estimation of the protein content with what is termed by Western definition prolonged lactation. By putting together a broad range of physiological, epidemiological, comparative, and ethnographic studies, this study confirms that while the nutritional content of human milk is variable, on average the milk protein concentration during the later stages of lactation is sensitive to the declining output of milk. This finding, together with the knowledge of the desired macronutrient intake for preterm infants, raises the question on whether excluding donors as early as six months postpartum is the correct strategy when setting limits on the duration of donation to milk banks.

It appears that the effect of weaning on milk protein content has not been adequately researched. A few research advances can be useful when thinking about next steps: protein content and composition from a representative sample of well-nourished women should be adjusted for parity, maternal health status, infant’s age and gender, daily milk output, gradual or rapid weaning, and biomarkers of tight junction regulation.

## Figures and Tables

**Table 1:** Search strategies and terms used to identify studies for this review.

**Search terms used to identify studies on milk protein content**
1. human milk AND protein AND content
2. lactation AND protein AND content
3. breastfeeding AND protein AND content
4. human milk AND macronutrients
5. lactation AND macronutrients
6. breastfeeding AND macronutrients
**Search terms used to identify factors associated with milk protein content**
(1) AND weaning
(2) AND weaning
(3) AND weaning
(5) AND extended
(6) AND extended

## References

[1] Valentine C.J., Morrow G., Reisinger A., Dingess K.A., Morrow A.L., Rogers L.K. (2017). Lactational stage of pasteurized human donor milk contributes to nutrient limitations for infants. Nutrients.

[2] Hartmann P.E., Kulski J.K. (1978). Changes in the composition of the mammary secretion of women after abrupt termination of breast feeding. J. Physiol..

[3] Neville M.C., Allen J.C., Archer P.C., Casey C.E., Seacat J., Keller R.P., Lutes V., Rasbach J., Neifert M. (1991). Studies in human lactation: Milk volume and nutrient composition during weaning and lactogenesis. Am. J. Clin. Nutr..

[4] Perrin M.T., Fogleman A.D., Newburg D.S., Allen J.C. (2017). A longitudinal study of human milk composition in the second year postpartum: Implications for human milk banking. Matern. Child. Nutr..

[5] Lönnerdal B., Erdmann P., Thakkar S.K., Sauser J., Destaillats F. (2017). Longitudinal evolution of true protein, amino acids and bioactive proteins in breast milk: A developmental perspective. J. Nutr. Biochem..

[6] Ballard O., Morrow A.L. (2013). Human milk composition: Nutrients and bioactive factors. Pediatr. Clin..

[7] Kreissl A., Zwiauer V., Repa A., Binder C., Thanhaeuser M., Jilma B., Berger A., Haiden N. (2016). Human Milk Analyser shows that the lactation period affects protein levels in preterm breastmilk. Acta. Paediatr..

[8] Czosnykowska-Lukacka M. Donor human milk after two years of lactation—Could it improve the macronutrients composition for preterm infants?. Proceedings of the 4th International Congress European Milk Bank Association (EMBA).

[9] Delgado C. (2013). Lactancia materna por dos o más años y su influencia en el crecimiento y desarrollo infantil: Una revisión sistemática. Cad. Saúde Pública.

[10] Dettwyler K.A. (1987). Breastfeeding and weaning in Mali: Cultural context and hard data. Soc. Sci. Med..

[11] Jelliffe D.B., Jelliffe E.F. (1978). The volume and composition of human milk in poorly nourished communities. A review. Am. J. Clin. Nutr..

[12] Nguyen D.A., Neville M.C. (1998). Tight junction regulation in the mammary gland. J. Mammary Gland Biol. Neoplasia.

[13] Tsugami Y., Matsunaga K., Suzuki T., Nishimura T., Kobayashi K. (2017). Phytoestrogens Weaken the Blood-Milk Barrier in Lactating Mammary Epithelial Cells by Affecting Tight Junctions and Cell Viability. J. Agric. Food. Chem..

[14] Kobayashi K., Tsugami Y., Matsunaga K., Oyama S., Kuki C., Kumura H. (2016). Prolactin and glucocorticoid signaling induces lactation-specific tight junctions concurrentwith β-casein expression in mammary epithelial cells. Biochim. Biophys. Acta.

[15] Widdows S.T., Lowenfeld M.F., Bond M., Shiskin C., Taylor E.I. (1935). A study of the antenatal secretion of the human mammary gland and a comparison between this and the secretion obtained directly after birth. Biochem. J..

[16] Linzell J.L., Peaker M. (1971). Intracellular concentrations of sodium, potassium and chloride in the lactating mammary gland and their relation to the secretory mechanism. J. Physiol..

[17] Garza C., Johnson C.A., Smith E.O., Nichols B.L. (1983). Changes in the nutrient composition of human milk during gradual weaning. Am. J. Clin. Nutr..

[18] Stelwgen K. (2001). Effect of Milking Frequency on Mammary Functioning and Shape of the Lactation Curve. J. Dairy Sci..

[19] Dallas D.C., Murray N.M., Gan J. (2015). Proteolytic systems in milk: Perspectives on the evolutionary function within the mammary gland and the infant. J. Mammary Gland Biol. Neoplasia.

[20] Prosser C.G., Saint L., Hartmann P.E. (1984). Mammary gland function during gradual weaning and early gestation in women. Aust. J. Exp. Biol. Med. Sci..

[21] Dewey K.G., Finley D.A., Lönnerdal B. (1984). Breast milk volume and composition during late lactation (7–20 months). J. Pediatr. Gastroenterol. Nutr..

[22] Lascelles A.K., Lee C.S., Larson B.L. (1978). Involution of the mammary gland. Lactation, a Comprehensive Treatise.

[23] Pagan J.D., Hintz H.F. (1986). Composition of milk from pony mares fed various levels of digestible energy. Cornell Vet..

[24] Jenness R. (1974). Biosynthesis and composition of milk. J. Investig. Dermatol..

[25] Konner M., Hewlett B.S., Lamb M.E. (2005). Hunter-gatherer infancy and childhood: The Kung and others. Hunter-Gatherer Childhoods: Evolutionary, Developmental and Cultural Perpectives.

[26] Barry H.I., Paxton L. (1971). Infancy and early childhood: Cross-cultural codes 2. Ethnology.

[27] Severn Nelson E.A., Schiefenhoevel W., Haimerl F. (2000). Child care practices in nonindustrial societies. Pediatrics.

[28] Tacail T., Thivichon-Prince B., Martin J.E., Charles C., Viriot L., Balter V. (2017). Assessing human weaning practices with calcium isotopes in tooth enamel. Proc. Natl. Acad. Sci. USA.

[29] Alston-Mills B., Iverson S., Thompson M. (2000). A comparison of the composition of milks from Meishan and crossbred pigs. Livestock Prod. Sci..

[30] Capuco A.V., Akers R.M. (2009). The origin and evolution of lactation. J. Biol..

[31] Millar J.S. (1977). Adaptive features of mammalian reproduction. Evolution.

[32] Hahn W.H., Jeong T., Park S., Song S., Kang N.M. (2018). Content fat and calorie of humanmilk is affected by interactions between maternal age and body mass index. J. Matern.-Fetal Neonatal Med..

[33] Yang T., Zhang Y., Ning Y., You L., Ma D., Zheng Y., Yang X., Li W., Wang J., Wang P. (2014). Breast milk macronutrient composition and the associated factors in urban Chinese mothers. Chin. Med. J..

[34] Rah J.H., Christian P., Shamim A.A., Arju U.T., Labrique A.B., Rashid M. (2008). Pregnancy and lactation hinder growth and nutritional status of adolescent girls in ruralBangladesh. J. Nutr..

[35] Hinde K. (2009). Richer milk for sons but more milk for daughters: Sex-biased investment during lactation varies with maternal life history in rhesus macaques. Am. J. Hum. Biol..

[36] Langer P. (2003). Lactation, weaning period, food quality, and digestive tractdifferentiations in eutheria. Evolution.

[37] Buss D.H. (1968). Gross composition and variation of the components of baboon milk during normal lactation. J. Nutr..

[38] Trott J.F., Simpson K.J., Moyle R.L., Hearn C.M., Shaw G., Nicholas K.R., Renfree M.B. (2003). Maternal regulation of milk composition, milk production, and pouch young development during lactation in the tammar wallaby (*Macropus eugenii*). Biol. Reprod..

[39] Beijers R.J., Graaf F.V., Schaafsma A., Siemensma A.D. (1992). Composition of premature breast-milk during lactation: Constant digestible protein content (as in full term milk). Early Hum. Dev..

[40] Ota K., Kimura M., Suzuki J. (1991). Lactation in the Japanese monkey (*Macaca fuscata*): Yield and composition of milk and nipple preference of the young. Primates.

[41] Roberts S.B., Cole T.J., Coward W.A. (1985). Lactational performance in relation to energy intake in the baboon. Am. J. Clin. Nutr..

[42] Contreras V.I.P., Bracamonte G.M.P., Bustamante L.A.L., Medina V.R.M., Rincón A.M.S. (2015). Milk composition and its relationship with weaning weight in Charolais cattle. Bras. J. Anim. Sci..

[43] Nicholas K.R., Hartmann P.E. (1991). Milk secretion in the rat: Progressive changes inmilk composition during lactation and weaning and the effect of diet. Comp. Biochem. Physiol. A Comp. Physiol..

[44] Piliena K., Jonkus D. (2013). Goat Milk Composition Variability after Kid Weaning. Agric. Sci..

[45] Boyce C., Watson M., Lazidis G., Reeve S., Dods K., Simmer K., McLeod G. (2016). Preterm human milk composition: A systematic literature review. Br. J. Nutr..

[46] Gidrewicz D.A., Fenton T.R. (2014). A systematic review and meta-analysis of the nutrient content of preterm and term breast milk. BMC Pediatr..

[47] Wojcik K.Y., Rechtman D.J., Lee M.L., Montoya A., Medo E.T. (2009). Macronutrient analysis of a nationwide sample of donor breast milk. J. Am. Diet. Assoc..

[48] Solien de Gonzales N.L. (1963). Breastfeeding, weaning and acculturation. J. Pediatr..

[49] Michaelsen K.F., Skafte L., Badsberg J.H., Jørgensen M. (1990). Variation in macronutrients in human bank milk: Influencing factors and implications for human milk banking. J. Pediatr. Gastroenterol. Nutr..

[50] Feng P., Gao M., Burgher A., Zhou T.H., Pramuk K. (2016). A nine-country study of the protein content and amino acid composition of mature human milk. Adv. Food Nutr. Res..

